# Interstitial 11q deletion: genomic characterization and neuropsychiatric follow up from early infancy to adolescence and literature review

**DOI:** 10.1186/1756-0500-7-248

**Published:** 2014-04-17

**Authors:** Renata Nacinovich, Nicoletta Villa, Serena Redaelli, Fiorenza Broggi, Monica Bomba, Patrizia Stoppa, Agnese Scatigno, Angelo Selicorni, Leda Dalprà, Francesca Neri

**Affiliations:** 1 Department of Surgery and Translational Medicine, University of Milan-Bicocca, Monza, Italy; 2 Childhood and Adolescence Neuropsychiatric Unit, San Gerardo Hospital, Monza, Italy; 3 Medical Genetics Laboratory, San Gerardo Hospital, Monza, Italy; 4 Ambulatorio Genetica Clinica Pediatrica, Clinica Pediatrica Università Milano Bicocca, Fondazione MBBM AO S, Gerardo Monza, Italy

**Keywords:** Chromosome 11q deletion, Array CGH, Genotype-phenotype correlation, Follow up, Rehabilitation programme

## Abstract

**Background:**

Interstitial deletions of chromosome 11 long arm are rarely observed and the associated phenotype ranges from normal to severe, depending on the position and size of the deletion and on the presence of unmasked recessive genes on the normal homologous. To our knowledge 32 cases are reported in literature with three family cases. Phenotype-genotype correlation is not very clear and the most common features are characteristic facial dysmorphisms, palate anomalies and developmental delay. Growth retardation is not typical and other major malformations are reported in some cases.

**Case Presentation:**

We described a child with 11q interstitial deletion diagnosed at birth with hypotonia and minor dysmorphisms using standard cytogenetic techniques; array CGH was subsequently performed to define the deletion at a molecular level.

**Conclusions:**

This case gave us the opportunity to attempt a genotype-phenotype correlation reviewing the literature and to describe a rehabilitative program that improved the development perspectives of this child.

## Background

Terminal 11q deletions are reported in literature to be associated with the well-described Jacobsen Syndrome. On the contrary interstitial deletions are very uncommon and heterogeneous in size and location of breakpoints, spanning from bands 11q13 to 11q23. From the first description by Taillemite et al. [1], 31 more cases were described [2-21], case ID 4366 in the European Cytogenetists Association Register of Unbalanced Chromosome Aberration, ECARUCA (http://www.ecaruca.net), case ID 3945 ECARUCA. Only in seven reports the deletion was characterized by cytogenetic molecular techniques [2-8]. The majority of deletions are de novo, but three family cases are described with normal or borderline phenotype [2,4,7]. Position and size of deletions are heterogeneous. The definition of the breakpoints of an interstitial 11q deletion by conventional techniques is difficult because the banding pattern could be confusing, but new molecular methods such as array CGH and SNPs allow an optimal definition of the breakpoint regions. Clinical phenotype includes several dysmorphic features, palate anomalies and developmental delay. Growth retardation, hypotonia, seizures, congenital heart malformation, kidney and skeletal anomalies are associated features in some cases.

Here we describe a case of de novo interstitial deletion of chromosome 11 long arm identified at birth while investigating mild dysmorphisms and hypotonia. The long term rehabilitative program is described and the good results obtained allow us to be positive in the prognosis of similar cases of 11q interstitial deletions. Finally we attempt a genotype/phenotype correlation reviewing the literature to search candidate genes with a role in the phenotype.

## Case presentation

The proband is a boy and he is the only child born from a 36 year old father and a 27 year old mother. The parents were healthy and unrelated; paternal family history was negative for genetic diseases, while in the maternal family, eight members were affected by retinitis pigmentosa with an autosomal dominant pattern of transmission. Prenatal ultrasonographic evaluation gave normal results in fetal growth and morphology. No prenatal invasive genetic test was performed.

The child was born by natural delivery after a 40 week gestation plus 5 days. Birth weight was 3420 g (25°- 50° centile for gestational age), length at birth was 52 cm, (between 50°- 75° centile), occipito-frontal circumference (OCF) was 35.5 cm (50°- 75° centile), APGAR score was 9/10. In neonatal period, the child presented hyperpyrexia (blood, urine and cerebrospinal fluid microbiological analyses were all negative) and greater than 10% weight loss, which regressed with adequate nutrition. Episodes of bronchospasm and tirage, repeated episodes of cyanosis and desaturation resolved spontaneously shortly before discharge (at one month of age). During childhood his height growth was at the lower limit. He did not show any major malformation, besides submucous cleft palate. Brain MRI scan did not show any structural anomalies.

He had mild myopia and his hearing was normal. He did not show any significant medical complications.

At the age of 12 years and 1 month his weight was 41.6 kg (25°-50° percentile), his height was 134.2 cm (<3° percentile) and his OFC was 53 cm (50° percentile). His Body Mass Index (BMI) was 23 (75°-90° percentile). Facial dysmorphic features included long face, high nasal bridge, short and smooth philtrum, micrognathia. He had small hands and nasal speech.

Endocrinological analysis (IGF-1 dosage and growth hormone stimulation test with arginine) showed results consistent with isolated growth hormone (GH) deficiency. A further brain MRI scan, carried out when he was twelve, revealed a small pituitary gland. GH treatment has been advised and the patient has just started this therapy.

Written informed consent was obtained from the parents for publication of this case report and any accompanying images.

At birth, karyotype on peripheral blood lymphocytes was requested because of mild hypotonia and minor dysmorphic features.

**Figure 1:**
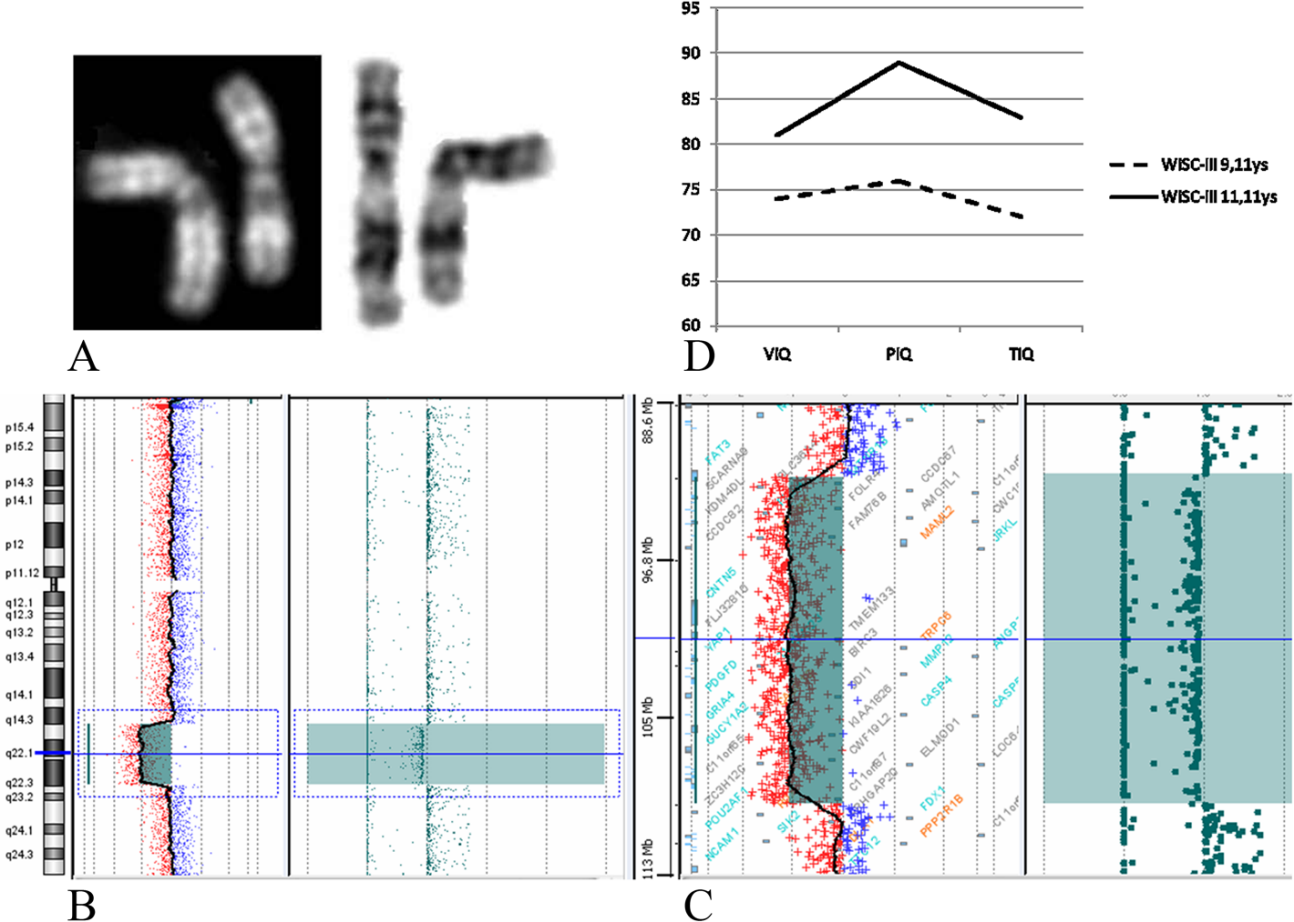
**Cytogenetic, arrayCGH and WISC-III results. (A)** QFQ and GTG banded chromosome 11 homologues. The deleted chromosome 11 is on the right. **(B)** Array-CGH analysis showing 17.2 Mb deletion (Log2ratio: −1.04) at 11q14.3q22.3 (nt 92434272–109584301; hg19, NCBI build 37) (light blue bar) and loss of heterozygosity with 337 single alleles SNP. **(C)** enlargement of the deleted region (CytoGenomics software Agilent). **(D)**: WISC-III results: Verbal(VIQ), Performance (PIQ) and Total IQ (TIQ) at 9.11 ys and after 2 years of rehabilitation and counselling to parents and teachers as regard as learning abilities.

Conventional cytogenetic analysis revealed an interstitial deletion on chromosome 11 from bands 11q13.5 to 11q21 (Figure 1A). The parents’ karyotypes were both normal and the analysis was extended to 100 metaphases to rule out 11q deletion mosaicism. At the age of twelve, a comparative genomic hybridization array (Array-CGH) was performed using a CGH + SNP 4 × 180 K microarray kit (Agilent Technologies). The microarray contained 110,172 probes with a median probe spacing of 25.3 Kb (5 KB in International Standards for Cytogenomic Arrays Consortium, ISCA) and 59,647 SNP (UCSC hg19, http://genome.ucsc.edu/). Array-CGH was performed according to the manufacturer’s instructions (Agilent Technologies) and allowed the identification of a more distal deletion of 17,2 Mb in size: from 11q14.3 to 11q22.3 (from nt 92,434,272 to nt 109,584,301)(555 probes with median Log2ratio value of −1.04). In the same region, 337 SNP probes showed a single allele (Figure 1B,C). No additional pathogenetic Copy Number Variations (CNVs) were detected. The molecular karyotype, defined following the International System of Chromosome Nomenclature 2013, was: arr 11q14.3q22.3(92,434,272-109,584,301)x1.

At 1 month of age, the proband’s neurological examination showed no major dysfunctions; clinical observation according to Prechtl´s Method on the Qualitative Assessment of General Movements [22] showed a pattern characterized by Poor Repertoire. His motor milestones were not significantly delayed: control of the head at 4 months, sitting without support at 8 months, hands and knees crawling at 11 months, standing with assistance at 13 months, walking alone at 24 months.

Parents reported vocalization, babbling and the first word “mamma” being uttered at one year of age. “Mamma” remained the only word used to name everything word until 19 months of age. After a period of hospitalization following an accidental fall which caused clavicle fracture and a second spell in hospital due to gastroenteritis, the child presented an arrest in speech and psychomotor competencies. These aspects were inscribed in a global regression of communicational and relational capacities.

Speech development between 20 and 36 months was characterized by: aphonia, amimia, pseudo- articulatory movements, reduced opening of the labial rima. Moreover, the child showed a poor communicative intent even at a non verbal level.

At 3 years he was a clumsy, passive and withdrawn child, not interested in playing. Therefore, at 3 years of age a psychomotor treatment with attention to relational aspects was introduced twice a week.

During the first year of rehabilitation, the proband reduced his more passive traits becoming more and more purposeful, actively responsive and assertive. Communicative intention emerged together with the increasing awareness of his speech difficulties.

There was a large discrepancy between the verbal comprehension (discrete) and expressive skills: he used gestures associated with vocalization to indicate, to formulate questions, to express disappointment or excitement. Then, speech therapy sessions began twice a week at the age of 4.

The proband showed a deficit of phonetic and non-phonetic phonation. Later, rhinolalia and rhinophonia, and a deficit in coordination and in phonological memory were observed. Consequently, a double diagnosis of specific expressive language disorder and of childhood apraxia of speech was formulated.

At 5 years, after one year of speech therapy, he achieved a quite complete phonetic repertoire, vocabulary increase with risk threshold for semantic errors, and emerging narrative skills. While he showed an adequate lexical comprehension, morphosyntactic comprehension was delayed of one year and communication was supported by mimics and gestures. At the same time, he developed social skills, showing more relational and playing capacities with peers and adults.

At age 6, verbal speech was sufficiently intelligible despite the persistence of hypotonic and uncoordinated glosso-velo-pharyngeal muscles.

His cognitive development, as measured by WIPPSI Scale, was in the lower limits of the normal range, with a discrepancy between verbal (VIQ) and performance (PIQ) scores (VIQ =85; PIQ =96; Full Scale IQ = 89) [23].

At the age of 8, the proband presented an impairment in the ability of naming objects and in lexical retrieval with the help of phonemic cues. On using the Italian version of the Peabody Picture Vocabulary Test [24], comprehension was almost adequate (Language quotient = 82).

Morphosyntactic comprehension using the Rustioni Metz test [25] showed a delay of about 2 years. The verbal short term memory span was 3 (inadequate).

Moreover, learning difficulties in the presence of a cognitive development at the lower limit of the normal range emerged. In particular, reading comprehension, measured using the MT battery of tests [26], was inadequate (correct answers: 6/10), in the absence of dyslexia and dysorthography, as measured with the Dyslexia and Orthographic coding test [27]. Therefore, the logopedic rehabilitation continued until the child was 9 years old, with the aim to improve oral and written language comprehension. In the meantime, his writing and reading abilities supported him in planning the phonological string in speech.

Afterwards, counselling to parents and teachers, together with neuropsychiatric follow up continued until today with longitudinal controls (twelve years). We considered highly important to support parents and teachers in finding a harmonious global development which could take into account not only the educational aspects, but also the emotional and relational sides.

We were able to observe a global improvement in cognitive abilities (as illustrated in Figure 1D, where WISC III at 9 and 11 years of age are compared) [28].

Currently the child shows good skills at school in the application of the learned mechanisms, but he has more difficulties when logic skills and problem solving abilities are required. However, he is able to evaluate his work and adopt compensatory strategies in order to overcome his difficulties. He is competitive with his schoolmates, comparing their tests results and scores. He shows a good personal and social independence and participates in curricular and extracurricular activities with peers.

## Conclusions

**Figure 2:**
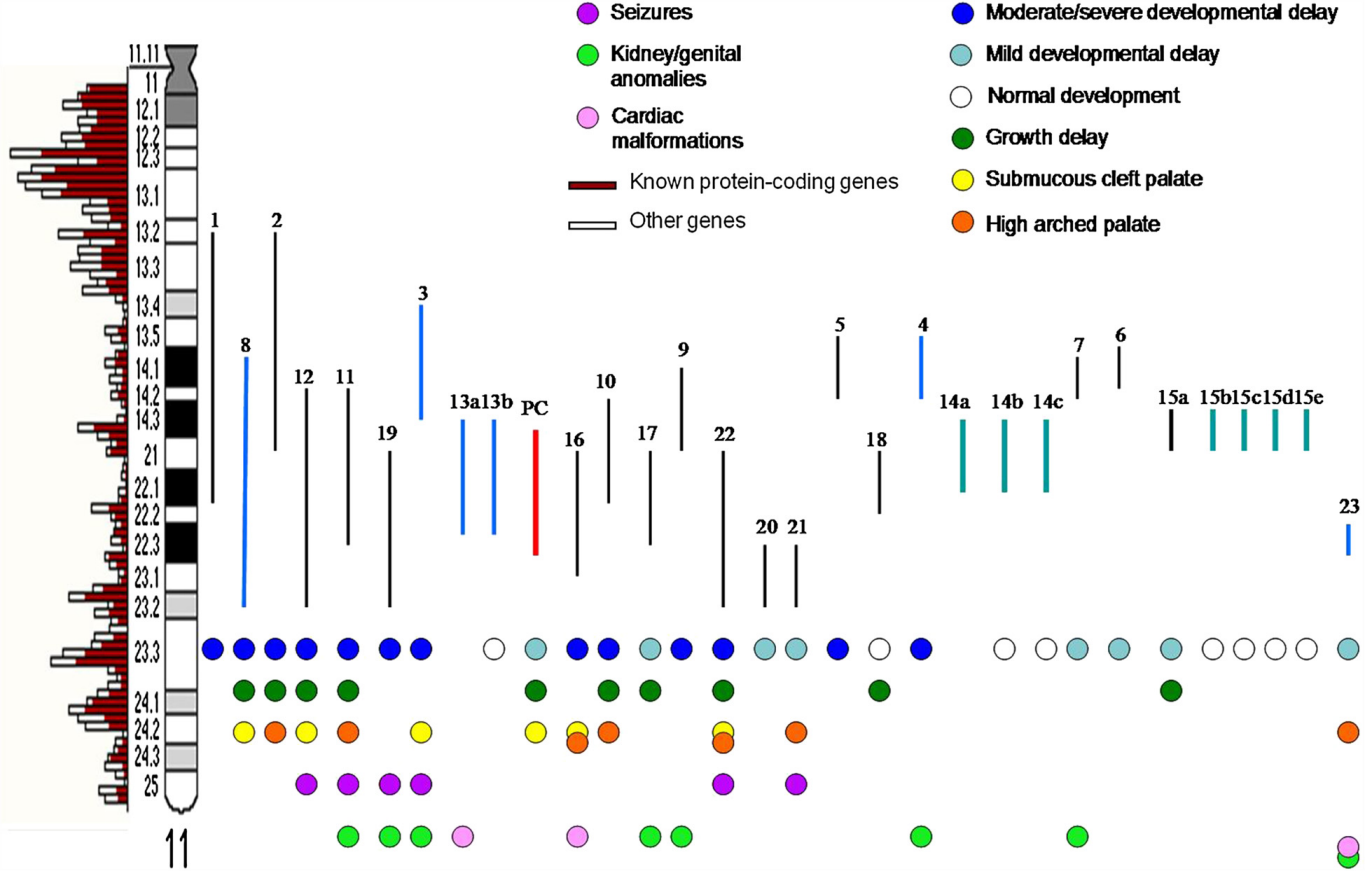
**Deletions map with major clinical signs reported as coloured dots.** Chromosome 11q arm ideogram with on the left gene density (from Ensemble http://www.ensembl.org/index.html) and, on the right, vertical bars are deletions reported in literature from the largest to the smallest. Red bar PC: present case. Green bars: family cases. Light blue bars: cases with molecularly defined deletions (13a and 13 b are family cases). The numbers above the bars correspond to those reported in Additional file 1: Table S1. Specific clinical signs are reported as coloured dots.

Interstitial 11q deletions are rare with only 32 patients described in literature to our knowledge [1-21] (Additional file 1: Table S1) they span from bands 11q13 to 11q23, but the majority of them has not been characterized with a molecular approach. A map was drawn up, in which deletions were positioned on the 11q chromosome ideogram (550 band resolution level) on the basis of the deletion sizes (from the largest to the smallest). When breakpoints were defined with a low resolution level (i.e. 11q13), we assumed the breakpoint to be in the middle (11q13.2). We disregarded two cases because of uncertain breakpoints [9,15]. We report some frequent clinical signs selected from Table 1. Heterogeneity in position and size is evident and a minimal common deleted region could not be identified (Figure 2). Moreover, cases with a molecularly-defined deletion (Figure 2, light blue bar) are only five [3,5-8]. Since they do not overlap and they are different in size a genotype/phenotype correl cannot be assessed. The first observation is that moderate/severe developmental delay correlates with the deletion size: in fact it was only reported in patients with larger loss, whereas growth retardation is distributed in the entire region. Growth is likely to be influenced by many factors, both genetic and environmental; it is therefore difficult to identify a close relationship with the deletion itself. Palate anomalies and seizures seem to correlate with more distal deletions (11q22-q23), while kidney/genital anomalies and cardiac malformations are reported in both proximal (11q13q21) and in distal ones (11q21q23.3) and don’t seem to correlate with the deletion size. The two families reported by Goumy et al. [2] and Li et al. [4] (Additional file 1: Table S1) with phenotypically normal deletion carriers are very interesting. Goumy et al. [2] describe a morphologically normal female fetus that inherited a 11q14.3q21.1 deletion from the mother whose clinical features were toe camptodactyly and ophtalmologic disorders. Her phenotypically normal maternal grandfather carried the same deletion. Prenatal diagnosis was carried out because of a positive Down Syndrome maternal serum screening. Li et al. [4] reported a 6 year old boy with mild intellectual impairment, short stature and a 11q14.3q21 deletion. Family study showed four males through three generations with the same deletion and apparently normal phenotype. The authors hypothesized a casual association between chromosomal anomaly and proband phenotype. In both reports, FISH with BAC clones was performed in order to partially define deletion breakpoints: Li identified a 3.6 Mb deletion (from rp11-792 M23 to rp11-573 M3) while Goumy defined the lowest deleted size of 8.5 Mb [from rp11-372E19 (91,733 Mb) to rp11-775E2 (100,424 Mb)]. These regions range approximately from nucleotide 89,255,000 (11q14.3) to 100,424,000 (11q22.1). Sparkes et al. [7] (Additional file 1: Table S1) described the third familial case: a male fetus tested for ultrasound multiple anomalies (choroid plexus cysts, echogenic intracardiac foci, a suspected structural cardiac malformation, left club foot and small cerebellum) with a maternally inherited 11q14.3q22.3 deletion. The mother was of normal intellect and healthy, but she had a surgically repaired bilateral club foot and high myopia. Moreover a cerebral MRI in the mother showed multifocal white matter changes and mild cerebral atrophy. Array CGH evidenced a 17,3 Mb deletion (from nt 89,492,818 to nt 106,832,040) and a 0,9 Mb duplication (from nt 88,258,744 to nt 89,103,489) in 11q21q23. From a molecular point of view, this case partially overlaps with our case (17,2 Mb deletion from nt 92,434,372 to nt 109,584,30), but we could not find any common clinical features: the case we are presenting showed mild developmental delay and submucous cleft palate; a cerebral MRI scan did not evidence any atrophy and he did not suffer from club foot.

**Table 1:** Summary of clinical features from the literature review of 32 cases and present case

**Deleted region (range)**	**Deletion size range (Mb)**	**Male/female**	**Age (range)**					
11q13-q23.2	0,743-35	13/18	prenatal-38yrs					
**Major malformation**		**Present case**	**Minor anomalies**		**Present case**	**Medical complications**		**Present case**
Trigonocephaly	4	-	Prominent forehead	4	-	Hypotonia	7	-
Dolichocephaly	2	-	Round face	2	-	Strabism	3	-
Brain anomalies	6	-	Uni/bilateral eyelids Ptosis	6	-	Myopia	2	+
Kidney anomalies	4	-	Up-slanted palpebral Fissures	7	-	Seizures	6	-
Submucous cleft palate	7	+	Periorbital fullness	3	-			
Heart defect	4	-	Epi/telecanthus	6	-	**Others**		
Genital anomalies	3	-	Hypertelorism	9	-	Microcephaly	3	-
Uni/bilateral club foot	4	-	Ears anomalies	13	-	Growth retardation	9	+
Iris and chorioretinal coloboma	2	-	Nose anomalies	12	+	Developmental delay	19	+
Retinal dysgenesis/bilateral exudative vitreoretinopathy	4	-	Full cheeks	4	-	Hyperactive behavior	3	-
			High arched palate	8	-	Sociable personality	4	-
			Mouth anomalies	11	-			
			Micro/retrognathia	9	+			
			Minor skeletal anomalies	12	-			

In the deleted region of our patient, about 94 genes are mapped [http://genome.ucsc.edu/]: 53 of them have known functions, some of them being associated with autosomal recessive disease, others have no OMIM phenotype. A biological role is still unkwnown for the other 41 genes.

Eight OMIM genes are expressed in the brain (FAT 3, MED17, PANX1, GPR83, CNTN5, KIAA1377,GRIA4 and GUCY1A2). The CNTN5 gene, coding for contactins which mediate cell surface interactions in the development of the nervous system, is described as been involved in autism spectrum disorders [29]. The GRIA4 gene belongs to the glutammate receptor family that plays an important role in excitatory synaptic transmission. Patients with mental retardation and multiple congenital abnormalities showed a significant copy number change in the glutammate receptor family [30]. Even if it is very difficult to correlate particular clinical features to a specific gene, we may hypothesize the role of haploinsufficiency of these two genes in the patient phenotype.

In literature [8,10,12,16,20] a psychomotor delay and a language disorder were described, in a few cases, as an expressive disorder. Our patient also showed the particular trait of a childhood apraxia of speech.

The phenotype we observed in our case has only been described in one other report [11] in which the proband shows poor eye contact and low interest in his surrounding. The rehabilitation process proposed and the neuropsychiatric follow up and counselling have allowed the recovery of adequate relational competences, improved speech and language abilities and a positive evolution of his global cognitive and adaptive capacities and social and school skills.

After considering all the collected clinical information, we drew the conclusion that it is not possible to define a distinctive phenotype of the 11q partial monosomy due to the heterogeneity in size and position of the deletions and to the absence of a minimal common deleted region. About family cases showing normal phenotype [2,4] we could hypothesize the effect of modifier genes or a compensatory gene expression of the allele on the normal chromosome 11. More literature data and a better molecular characterization are needed to confirm this hypothesis. Moreover, the phenotype is not only due to haploinsufficient genes but it is the result of complex gene – gene and gene-environment interactions.

We found this case particularly interesting because it gave us the opportunity to describe a rehabilitation program that we believe has improved the development perspectives of this child and therefore his quality of life.

## Consent

Written informed consent was obtained from the patient’s parents for publication of this Case Report and any accompanying images. A copy of the written consent is available for review by the Editor-in-Chief of this journal.

## Competing interests

The authors do not have any competing interest, financial or otherwise, to declare.

## Authors’ contributions

RN, FB contributed to design the study, collected the literature data and performed the neuropsychiatric evaluation of the patient and the rehabilitation programme and wrote a part of the article MB contributed to design the study, collected the literature data and wrote a part of the article. PS contributed to the neuropsychiatric evaluation of the patient. NV contributed to design the study, performed genetic studies on the patient, collected the literature data and wrote a part of the article. S.R. performed arrayCGH. LD and FN contributed to design the study and critically read the manuscript. AS, AS performed the paediatric evaluation of the patient. All authors read and approved the final manuscript.

## Supplementary Material

Additional file 1 Literature review of the 11q interstitial deletions from proximal to distal deletion. Click here for file
