# Supplementary material for: Interstitial 11q deletion: genomic characterization and neuropsychiatric follow up from early infancy to adolescence and literature review
Source: BMC Res Notes. 2014 Apr 17;7:248. doi: 10.1186/1756-0500-7-248 (PMC4108053; doi:10.1186/1756-0500-7-248)
Supplement: Additional file 1 — Literature review of the 11q interstitial deletions from proximal to distal deletion. [file 1756-0500-7-248-S1.doc]

**Table S1 : literature review of the 11q interstitial deletions from proximal to distal deletion**

|  | **Taillemite**  **1** | **Guć-Sćekić**  **2** | **Wincent 3** | **Melis 4** | **Joce**  **5** | **ecaruca° ID 4366**  **6** | **ecaruca° ID 3945**  **7** | **Li 2006***  **8** | **Stratton 9** | **Wakazono 10** |
| --- | --- | --- | --- | --- | --- | --- | --- | --- | --- | --- |
| deleted region | 11q13-q22 | 11q13-q21 | 11q13.4-q14.3 | 11q13.5-q14.2 | 11q13.5-q14.2 | 11q14.1 | 11q14.1-q14.2 | 11q14.1-q23.2 | 11q14.1-q21 | 11q14-q22 |
| deleted region by molecular techniques |  |  | 72,376,209 -90,593,248 | 75,648,036-86,462,174 hg18 |  |  |  | 77,64-113,52 UCSC may 2004 |  |  |
| deletion size (Mb) |  |  | 18,2 | 10,8 |  |  |  | 35 |  |  |
| sex |  | male | male | male | male | male | male | female | female | female |
| age (years) |  | 0.2 | 3.5 | 5 | 6 | 6 | 2.5 | 3 | 4 | 1 |
| growth retardation |  | + |  | - |  |  |  | + | - | + |
| microcephaly |  |  | + |  | - |  |  | + |  |  |
| trigonocephaly |  | - |  |  |  |  |  |  | + |  |
| dolichocephaly |  |  |  |  |  |  |  |  |  | + |
| brain anomalies |  |  | - | - |  |  |  | - |  |  |
| kidney anomalies |  |  |  |  |  |  |  |  | + |  |
| submucous cleft palate |  |  | + |  | - |  |  | + | - |  |
| heart defect |  |  |  | tricuspid insufficiency | - |  |  | - |  |  |
| genital anomalies |  |  | + | + |  |  | + |  |  |  |
| uni/bilateral club foot |  |  |  |  |  |  |  |  |  |  |
| iris and chorioretinal coloboma |  |  |  | + |  |  |  |  |  |  |
| retinal dysgenesis/bilateral exudative vitreoretinopathy |  |  |  |  |  |  |  | + |  |  |
| prominent forehead |  | + |  |  |  |  |  |  |  |  |
| round face |  |  | + | + |  |  |  |  |  |  |
| uni/bilateral eyelids ptosis |  |  | + | + |  | + |  |  |  | + |
| up-slanted palpebral fissures |  | - |  | + |  | + | + | + |  |  |
| periorbital fullness |  |  |  | + | + |  | + |  |  |  |
| epi/telecanthus |  | + | + |  |  | + |  |  |  | + |
| hypertelorism |  | + |  | + |  |  |  |  |  |  |
| ears anomalies |  | + | + | + | + | + | + |  | - | + |
| broad and/or flat nasal bridge |  |  | + | + | - | + | flared nares | anteverted nares | - | + |
| full cheeks |  |  | + | + | + |  | + |  |  |  |
| high arched palate |  | + |  |  |  |  |  |  |  | + |
| mouth anomalies |  |  | + | + | + | + | + |  |  | + |
| micro/retrognathia |  | + |  |  | + | + |  | + |  | + |
| minor skeletal anomalies |  | + | - | + |  | + |  | + |  |  |
| hypotonia |  |  | + |  |  | + |  | + |  |  |
| strabism |  |  | + |  |  |  |  |  |  |  |
| myopia |  |  |  |  |  |  |  |  |  |  |
| seizures |  |  | + | - |  |  |  |  |  |  |
| developmental delay | + | + | moderate | moderate | moderate | mild | mild | + | moderate | + |
| hyperactive behavior |  |  | + | + | - |  |  | + |  |  |
| sociable personality |  |  | + | + | + |  |  |  |  |  |

* complex karyotype: del(16)(q22.3) 1,14 Mb; balanced t(5;8)

° www.ecaruca.net

|  | **Sachdeva 11** | **Ono**  **12** | **present case (PC)** | **Sparkes 13a** | **Sparkes** 13b** | **Goumy 14a** | **Goumy 14 b,c** | **Li 2002 15a** | **Li 2002 15b,c,d,e** | **Meyer 16** |
| --- | --- | --- | --- | --- | --- | --- | --- | --- | --- | --- |
| deleted region | 11q14.2-q22.3 | 11q14.2-q23.2 | 11q14.3-q22.3 | 11q14.3-q22.3mat | 11q14.3q22.3 | 11q14.3-q22.1 mat | 11q14.3-q22.1pat | 11q14.3-q21 familial | 11q14.3-q21 familial | 11q21-q23.1 |
| deleted region by molecular tecniques |  |  | 92,434,372-109,584,301 |  | 89492819-106832038 NCBI 36 |  |  |  |  |  |
| deletion size (Mb) |  |  | 17,2 |  | 17,3 | 8,5 - 16 (BAC) | 8,5 - 16 (BAC) | 3,6 | 3,6 |  |
| sex | female | female | male | male fetus | female | female fetus | mother and grandfather | male | four males | female |
| age (years) | 16 | 11 | 12 | pregnancy (TAB) | 38 | prenatal | adults | 6 | adults | 21 |
| growth retardation | + | + | + |  |  |  |  | + | - |  |
| microcephaly | + | - | - |  |  |  |  | - | - |  |
| trigonocephaly | + | - | - |  |  |  |  | - | - | - |
| dolichocephaly |  |  | - |  |  |  |  | - | - |  |
| brain anomalies | + | - | - | + | + |  |  | - | - |  |
| kidney anomalies | + |  | - | - | - |  |  | - | - |  |
| submucous cleft palate |  | + | + |  |  |  |  | - | - | + |
| heart defect |  | - | - | + |  |  |  | - | - | + |
| genital anomalies |  |  | - |  |  |  |  | - | - |  |
| uni/bilateral club foot | + |  | - | + | + |  |  | - | - |  |
| iris and chorioretinal coloboma |  | - | - |  |  |  |  | - | - |  |
| retinal dysgenesis/bilateral exudative vitreoretinopathy | + | + | - |  |  |  |  | - | - |  |
| prominent forehead |  | - | - |  |  |  |  | - | - | + |
| round face |  |  | - |  |  |  |  | - | - |  |
| uni/bilateral eyelids ptosis | - | - | - |  |  |  |  | - | - |  |
| up-slanted palpebral fissures | + |  | - |  |  |  |  | - | - |  |
| periorbital fullness |  |  | - |  |  |  |  | - | - |  |
| epi/telecanthus | - | + | - |  |  |  |  | - | - |  |
| hypertelorism | - | - | - | + | + |  |  | - | - |  |
| ears anomalies |  | - | - |  |  |  |  | - | - | + |
| broad and/or flat nasal bridge | + | + | + |  |  |  |  | - | - | + |
| full cheeks |  |  |  |  |  |  |  | - | - |  |
| high arched palate | + | - |  |  |  |  |  | - | - | + |
| mouth anomalies |  | - | - |  |  |  |  | - | - | + |
| micro/retrognathia | + | - | + |  |  |  |  | - | - | + |
| minor skeletal anomalies | + |  | - |  |  |  | +/- | - | - |  |
| hypotonia |  |  | - |  |  |  |  | - | - |  |
| strabism | + |  | - |  |  |  | +/- | - | - |  |
| myopia | + |  | + |  | + |  |  | - | - |  |
| seizures | + | + | - |  |  |  |  | - | - |  |
| developmental delay | severe | + | + |  | - |  |  | mild | - | + |
| hyperactive behavior |  |  |  |  |  |  |  | attention disorder | - |  |
| sociable personality |  |  |  |  |  |  |  | - | - |  |

** dup11q21q23 (88258744-89103489):0,9 Mb

|  | **Horelli-Kuitunen**  **17** | **Ikegawa*** 18** | **Ono 19** | **Syrrou 20** | **De Pater 21** | **Ono 22** | **Krgovic 23** | **Carnevale**  **24** | **Klep-de-Pater**  **25** |
| --- | --- | --- | --- | --- | --- | --- | --- | --- | --- |
| deleted region | 11q21-q22.3 | 11q21-q22.2 | 11q21-q23.2 | 11q22.3-23.2 | 11q22.3-q23.2 | 11q21-q23 | 11q22.3 | 11q13-q21 or 11q21-q23 | 11q13-q21 or 11q21-q23 |
| deleted region by molecular tecniques |  |  |  | YAC878C12+-MLL- |  |  | 107349817-108093259 |  |  |
| deletion size (Mb) | 20 cM | 0,8-7,3 |  |  |  |  | 0,743 |  |  |
| sex | female | male | male | male | male | male | female | female | female |
| age (years) | 3 | 15 | 12 | 2,8 | 21 | 8 | 5 |  | 7 |
| growth retardation | + | + | - | - | - | + |  | - | - |
| microcephaly |  | - | - | relative |  | - |  |  | - |
| trigonocephaly | + | - | - | mild | - | - |  |  | - |
| dolichocephaly |  | - |  |  |  |  |  | + | - |
| brain anomalies | + |  | - | - |  | + | + |  | - |
| kidney anomalies | + |  |  |  |  |  | + |  | - |
| submucous cleft palate |  | - | - | - |  | + |  | + | + |
| heart defect |  |  | - |  | - | - | + | + | - |
| genital anomalies |  |  |  |  |  |  |  |  | - |
| uni/bilateral club foot | + |  |  |  |  |  |  |  | - |
| iris and chorioretinal coloboma |  | - | + | - |  | - |  |  | - |
| retinal dysgenesis/bilateral exudative vitreoretinopathy |  |  | + | - |  | - |  |  | - |
| prominent forehead |  | - | - | - |  | - | + | + | - |
| round face |  | - |  |  |  |  |  |  |  |
| uni/bilateral eyelids ptosis |  | - | - |  |  | - | + |  | + |
| up-slanted palpebral fissures |  | - |  |  | - |  | + | + | - |
| periorbital fullness |  | - |  |  |  |  |  |  | - |
| epi/telecanthus |  | - | - | - | - | - |  | + | - |
| hypertelorism | + | - | + | - | - | + | + |  | + |
| ears anomalies | + | - | - | + | + | + | + | - | - |
| broad and/or flat nasal bridge |  | - | - | - | - | - | small nose | + | + |
| full cheeks |  | - |  |  |  |  |  |  |  |
| high arched palate |  | - | - | - | + | + | + |  | + |
| mouth anomalies |  | - | - | + |  | + | + |  | + |
| micro/retrognathia |  | - | - |  | - | + |  | + | - |
| minor skeletal anomalies |  | + |  | + | + |  | + | + | + |
| hypotonia | + |  |  | mild |  |  | + | - | + |
| strabism |  | - |  | - |  |  |  |  | - |
| myopia |  |  |  | - |  |  |  |  | - |
| seizures |  |  | + |  | + | + | - |  | - |
| developmental delay | mild | normal | + | borderline | + | + | mild | normal | mild |
| hyperactive behavior |  |  |  |  |  |  | - |  |  |
| sociable personality |  |  |  |  |  |  | + |  |  |

*** Pseudoachondroplasia and heterozigous COMP mutation
